# Synthesis and Performance Evaluation of a New Deoiling Agent for Treatment of Waste Oil-Based Drilling Fluids

**DOI:** 10.1155/2014/852503

**Published:** 2014-06-22

**Authors:** Pingting Liu, Zhiyu Huang, Hao Deng, Rongsha Wang, Shuixiang Xie

**Affiliations:** ^1^College of Chemistry and Chemical Engineering, Southwest Petroleum University, Chengdu 610500, China; ^2^CNPC Research Institute of Safety & Environment Technology, Beijing 102206, China

## Abstract

Oil-based drilling fluid is used more and more in the field of oil and gas exploration. However, because of unrecyclable treating agent and hard treatment conditions, the traditional treating technologies of waste oil-based drilling fluid have some defects, such as waste of resource, bulky equipment, complex treatment processes, and low oil recovery rate. In this work, switchable deoiling agent (SDA), as a novel surfactant for treatment of waste oil-based drilling fluid, was synthesized by amine, formic acid, and formaldehyde solution. With this agent, the waste oil-based drilling fluid can be treated without complex process and expensive equipment. Furthermore, the agent used in the treatment can be recycled, which reduces waste of resource and energy. The switch performance, deoiling performance, structural characterization, and mechanisms of action are studied. The experimental results show that the oil content of the recycled oil is higher than 96% and more than 93% oil in waste oil-based drilling fluid can be recycled. The oil content of the solid residues of deoiling is less than 3%.

## 1. Introduction

Petroleum and natural gas are so important strategic resources that it is necessary to exploit them for all the countries. However, the large amount of waste drilling fluid, especially the waste oil-based drilling fluid which is produced in the process of drilling during field development [1, 2], is very harmful to the environment [3, 4]. Waste oil-based drilling fluid is classified as hazardous waste for the reason that it contains a lot of oil, heavy metals, and organic pollutants [5, 6]. Therefore, waste oil-based drilling fluid must be treated properly, or it will cause great harm to environment, animals and human [7, 8].

Because of the high oil content and stable emulsion of waste oil-based drilling fluid, treatment of it is different from that of the other drilling fluid and a great challenge [9]. If the oil in waste oil-based drilling fluid cannot be recycled, harmless processing will be very difficult to realize and the oil will be wasted [10]. Though some treatment technologies [11], such as thermal desorption, microwave processing, solvent extraction, chemical demulsification, and supercritical fluid extraction, have been used to treat the waste oil-based drilling fluid, it have been proved that all of them have some disadvantage [12–14]. Thermal desorption technology requires expensive equipment and high temperature, which causes high cost and energy consumption [15–17]. Microwave processing [18–20], which is an alternative to thermal desorption, also requires complex equipment and causes high cost. Solvent extraction technology and chemical demulsification technology require unrecyclable solvent or reagent to be added in the processing, which cause waste of resource [21, 22]. Though supercritical fluid is reusable, supercritical fluid extraction technology needs high temperature and high pressure [23, 24]. Therefore, it is very significant to develop more economic and effective methods to deal with waste oil-based drilling fluid.

In this paper, a novel surfactant for treatment of waste oil-based drilling fluid, called switchable deoiling agent (SDA), is synthesized to solve the problems of unrecyclable treating agent and complex processing condition. SDA is able to treat the waste oil-based drilling fluid without complex process and expensive equipment. Furthermore, SDA used in the treatment can be recycled, which overcomes the defect that the traditional agent can be used only once. Therefore, it is significant to simplify the processes and reduce waste of resource and energy by the method of using SDA.

## 2. Materials and Methods

### 2.1. Materials

Sodium hydroxide, potassium hydroxide, sodium chloride, sodium sulfate, and hydrochloric acid were purchased from Beijing Chemical Works. Organic amine was purchased from Sinopharm Chemical Reagent Co., Ltd., China. Formaldehyde, formic acid, and tetrachloromethane were purchased from Xilong Chemical Co., Ltd., China. All the reagents mentioned above were of analytical reagent grade and used without further purification. Switchable deoiling agent for waste oil-based drilling fluid was synthesized. Waste oil-based drilling fluid was supplied by Daqing Oilfield in China.

### 2.2. Methods

#### 2.2.1. Synthesis of SDA

A magnetic stirrer rotor was put into a two-neck bottle and kept rotating. Amine, formic acid, and formaldehyde solution were added in turns with the molar ratio of 1 : 5 : 2. When fog happens, two-neck bottle was cooled by ice packs to maintain relatively low temperature. Then, condenser tube was installed on the vertical neck of the two-neck bottle and temperature probe was installed on the other neck. The temperature was maintained at 100°C for 3 h. Heating was stopped when the mixture gradually changes from light yellow to dark brown. Then, hydrochloric acid (HCl) with the same molar as amine was added when the bottle was cooled to room temperature. Heating was restarted and kept until the extra formic acid and formaldehyde were distilled out. Then, 30% sodium hydroxide (NaOH) solution was added into the two-neck bottle to adjust the pH of the liquid to 7-8 (the color of the solution would fade from dark brown to yellow). After that, the distillation equipment was installed to distill the mixture until the liquid in the bottle turns to red. After liquid distilled out was separated, a small amount of potassium hydroxide (KOH) was added to break emulsion. The separated upper layer liquid is the target product.

#### 2.2.2. Pass Gas Through

**Figure 1:**
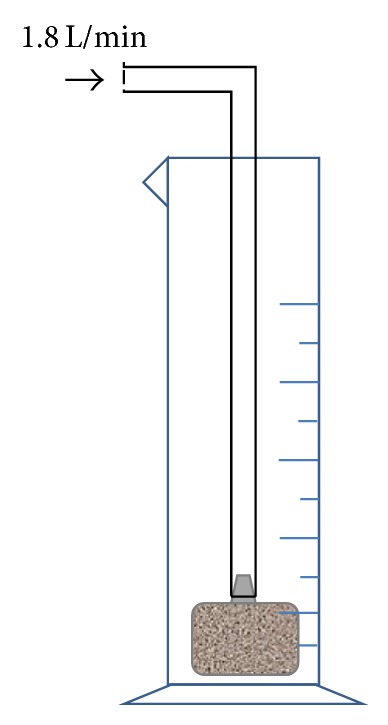
Diagram of ventilation device.

The ventilation device (as shown in Figure 1) used to pump gas into mixed liquid consists of 2 parts: a 100 mL cylinder and a 10 mm diameter aeration head connected with a 4 mm diameter flexible pipe. The gas was passed through at the speed of 1.8 L/min through the pipe and aeration head. Due to its own weight, the aeration head always remains at the bottom of the cylinder, which produces uniform and fine bubbles in the liquid and makes intensive mixture of gas and liquid.

#### 2.2.3. Determination of Oil Content in Waste Oil-Based Drilling Fluid

The water content determination apparatus, constituted by a condenser-west tube, a receiver, and the round bottomed flask, is used in this experiment. First, about 10 g (with accuracy of ±0.1 g) waste oil-based drilling fluid sample and 50 mL anhydrous petroleum ether (90–120°C) were added into the round bottomed flask. Then, the condenser-west tube and receiver were connected and the flask was heated. During the whole process, reflux rate was controlled at 2–4 drops per second. Heating was stopped when the volume of water in the receiver is no longer increased. Then, the volume of the water in the receiver was recorded. When the temperature was low enough, the rest in the flask was cleaned with anhydrous petroleum ether and filtrated with Buchner funnel. Finally, the filter residue was weighted after it was dried at the temperature of 105°C. The oil content was calculated according to the following formulas:

(1) $$\begin{array}{c} H=\frac{V_{w}\times \rho_{w}}{W}\times 100\%,\\ S=\frac{V_{f}}{W}\times 100\%,\\ O=1-H-S, \end{array}$$

where *H* represents the rate of water content, %; *O* represents the rate of oil content, %; *S* denotes the rate of solid content, %; *W* denotes the weight of the sample (g); *V*_*w*_ is the volume of distillate (mL); *ρ*_*w*_ is the density of water (g/mL); *W*_*f*_is the weight of filter residue (g).

#### 2.2.4. Determination of Oil Content in Wastewater and Solid Waste

*(A) Preprocessing.* First, 10 mL acidulated water sample was mixed fully with 10 g sodium chloride (NaCl) and 20 mL tetrachloromethane (CCl_4_) in a separating funnel. Then, the under-layer liquid was filtrated by a sand core funnel with 1 cm anhydrous sodium sulfate on the top. The filtrate was collected in a 50 mL volumetric flask. After that, 20 mL CCl_4_ was added for the second extraction. Finally, the sand core funnel was cleaned with a small amount of CCl_4_ and additional CCl_4_ was added into the volumetric flask to the volume of 50 ml.

25 mL CCl_4_ was added into the mixed liquid which was fully mixed with 1.00 g solid residue and 20.00 g anhydrous sodium sulfate. Then, the mixed liquid was filtrated by a sand core funnel. The filtrate was diluted to 25 mL with CCl_4_ after the sand core funnel was cleaned twice with CCl_4_. At last, 1 mL liquid was drawn out and diluted to 50 mL.

*(B) Determination by Infrared Spectrophotometry.* The oil content of liquid in 50 mL flasks was determined successively with infrared oil analyzer. The data obtained by infrared oil analyzer was multiplied by dilute multiple to obtain the oil content of oily wastewater and oily solid waste.

#### 2.2.5. Structural Characterization

Fourier transform infrared spectroscopy (FTIR) (Nicolet iS50) was used for structural characterization of the deoiling agent. The samples were prepared based on pure potassium bromide (KBr) discs. First, the fully dried KBr was grinded to below 2 *µ*m with agate mortar. After that, 70 mg grinded KBr was weighed and put into specific tableting press and then pressed to homogeneous transparent round slice under the pressure of 10 t with 5 min. Then, the pure KBr discs were impregnated into the sample solution for seconds. After that, the discs were taken out and the excessive samples were absorbed by filter paper. Finally, the prepared samples were determined with infrared spectrometer on the sample holder.

## 3. Results and Discussion

### 3.1. Contamination in Waste Oil-Based Drilling Fluid

Five kinds of waste oil-based drilling fluid from Daqing Oilfield were used to analyze what the key components and the main pollutants were in.

As shown in Tables 1 and 2, the oil content of these waste oil-based drilling fluids is between 26.7% and 39.6%. The primary pollutant is oil. Therefore, removing and recycling the oil is the most important target for harmless treatment and resource utilization of waste oil-based drilling fluid.

### 3.2. Performance of SDA

#### 3.2.1. Switch Performance of SDA

**Figure 2:**
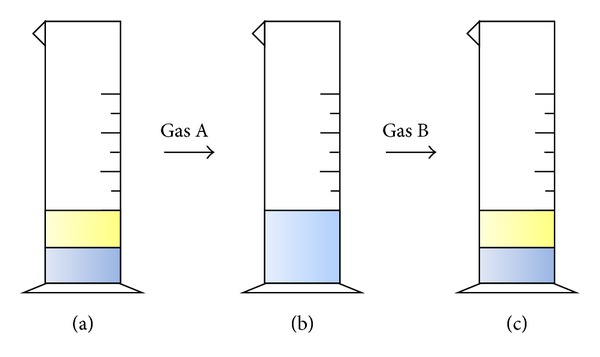
Switch processes.

*(A) Switch Processes*. The main characteristic of the deoiling agent is that its hydrophilicity can be converted. Normally, the deoiling agent is hydrophobic and stratification is obvious when it is mixed with water (Figure 2(a)). However, after gas A (CO_2_) is bubbled into the mixtures, the deoiling agent becomes hydrophilic and the solution is homogeneous (Figure 2(b)). Again, the deoiling agent becomes hydrophobic and stratification is obvious after gas B (air, Ar, or N_2_) is passed through (Figure 2(c)).

*(B) Switch from Hydrophobicity to Hydrophilicity. *The experiment of switch from hydrophobicity to hydrophilicity was done under the condition of 22°C and 25% RH. First, 10 mL deoiling agent was mixed with 10 mL pure water, which formed layered liquid. Then, gas A was passed through the liquid above.  Table 3 shows the time of passing gas through, volume of the hydrophilic layer, volume of the hydrophobic layer, and volume of switched deoiling agent. The conversion rate was calculated based on these results. Gas was stopped when the liquid changed to be homogenous phase, which meant that the deoiling agent was switched to be hydrophilic. In the homogenous phase, the liquid-liquid interface disappeared and the mixed liquid became slightly translucent white liquid. When deionized water was added to the uniform liquid, it also remained miscible.

**Figure 3:**
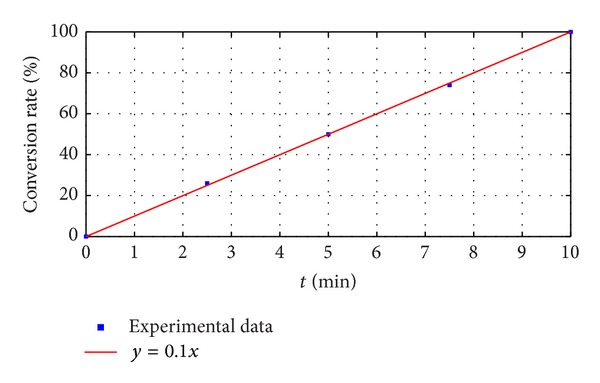
The fitting curve of the relationship between conversion rate of SDA and the time of passing gas A through.

Figure 3 shows the relationship between conversion rate of SDA (*S*) and the time (*t*) of passing gas A through. The blue points are experiment data and the red line is the fitting curve. The function of the fitting curve is *y* = *x*, where *y* is conversion rate (*S*) and *x* is the time (*t*). That means that, when gas A is passed through at the speed of 1.8 L/min, the switch speed is 10%/min. Finally, the conversion rate rises to 100% after passing gas through for 10 min. Therefore, there is no loss in the switch process.

*(C) Switch from Hydrophilicity to Hydrophobicity*. The experiment of switch from hydrophilicity to hydrophobicity was done under the condition of 22°C and 25% RH. Gas B was passed into the mixed phase (prepared in Section 2.2.1) until the volume of hydrophobic layer stopped increasing.  Table 4 shows the time of passing gas B through and the corresponding volume of hydrophobic layer.

**Figure 4:**
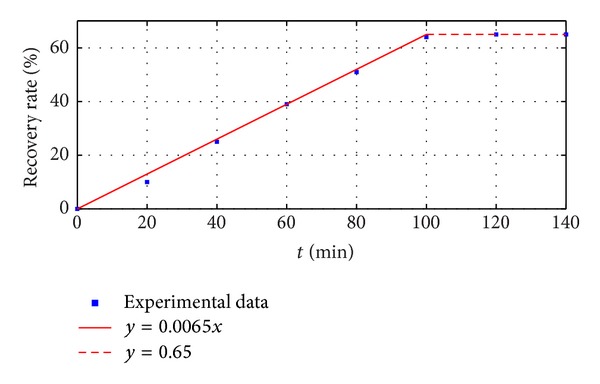
The fitting curve of the relationship between recovery rate of SDA and the time of passing gas A through.

As shown in Table 4, the volume of oil layer increases very slightly during 100–120 min and has no change during 120–140 min, which means that almost all of the deoiling agent is switched within 120 min.  Figure 4 shows the fitting curve of the recorded experiment data between 0 min and 140 min. The results show the relationship recovery rate of SDA and the time of passing gas B through. The function of the fitting curve is *y* = 0.65*x*  (0 < *x* < 100), *y* = 65  (*x* ≥ 100), where *y* is recovery rate (*R*) and *x* is the time (*t*). This means that, when gas B is passed through at the speed of 1.8 L/min, the speed of switch is 0.65%/min.

In summary, the deoiling agent is hydrophobic in normal state and shows poor solubility in water. When gas A is passed through, the deoiling agent is switched to hydrophilicity and shows high water solubility. When gas B is passed through, the deoiling agent is switched back to hydrophobicity. The switching rate of deoiling agent from hydrophobicity to hydrophilicity is 15 times faster than that from hydrophobicity to hydrophilicity. Both relationships between the switched volume and the time of passing gas through in these two processes can be defined as a function *y* = *kx*, which means that the volume is proportional to the time. The experiments and analysis above show that the speed of switch from hydrophobicity to hydrophilicity is fast and the loss ratio is nearly 0%. However, the speed of switch from hydrophobicity to hydrophilicity is slow and the loss ratio is up to 35%. Therefore, there will be important researches in our future work for improving the recycle technology to reduce the loss and increasing the speed of switch from hydrophobicity to hydrophilicity.

#### 3.2.2. Deoiling Performance of SDA

**Figure 5:**
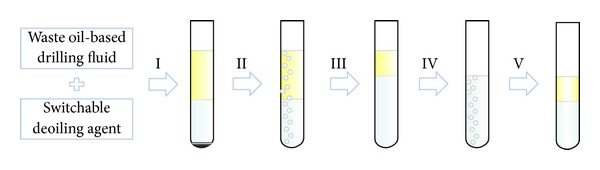
The process of oil removal and SDA recycle.

Figure 5 shows the process switchable deoiling agent used for removing oil from waste oil-based drilling fluid. In the first step, waste oil-based drilling fluid and SDA were mixed intensively and then stirred. The mixture was gradually divided into three layers: the upper layer was the hydrophobic layer, the mixture of SDA and oil; the intermediate layer was water; the lower layer was solid precipitation. In the second step, the solid precipitation was filtered and then gas A was passed through to switch SDA from hydrophobic to hydrophilic. In the third step, gas A was passed through continually until the layer stopped rising, which indicated that almost all of SDA was switched to hydrophilic and the oil was separated. In the fourth step, the oil was recycled and gas B was then passed through the remaining liquid. SDA was gradually switched to hydrophobic and floated upwards as oil droplet. In the fifth step, gas B was passed through until the volume of the upper layer stopped increasing. At this moment, almost all of SDA was switched back to hydrophobic and separated from the water. These SDA could be reused to deoil.

Sample 1 as an example was used to evaluate treatment effect. First, 41.5 g sample 1 and 100 mL switchable deoiling agent were mixed sufficiently. The mixture was sitting until clear hierarchy appeared. Solid precipitate was isolated after that. Then, 200 mL water was added into the mixture while gas A was continuously passed though. After that, the gas was shut down five min later when the bottom of the mixture was clear and droplets was flowed with gas A. At last, the separated upper black oil layer was weighed after the mixture was sat for 30 min or centrifuged for 5 min at the speed of 3000 r/min. The oil phase weighed 10.68 g. The rest of transparent mixture gradually separated into two layers after passing gas B though aqueous layer. Gas B was passed through until the volume of upper layer stopped increasing. The liquid in upper layer is recyclable switchable deoiling agent, and the lower is oily wastewater. It is found that the recycled agent can have the same nature with the new agent. The oil content of oily wastewater and oily solid waste was detected finally. The treatment effect of sample 1 was calculated as follows.

The oil content of extracted oil was calculated according to the following equations:

(2) $$\begin{array}{c} P=1-\frac{V_{w}\times \rho_{w}}{W}\times 100\%-\frac{W_{f}}{W}\times 100\%, \end{array}$$

where *P* is the oil content of sample 1, %; *W* is the quality (g); *V*_*w*_ is the volume of distilled water (mL); *ρ*_*w*_ is the density of water (g/mL); *W*_*f*_ is the density of residue (g).

The percentage of oil recycle was calculated as follows:

(3) $$\begin{array}{c} R=\frac{W_{oe}\times P_{oe}}{W_{om}\times P_{om}}\times 100\%, \end{array}$$

where *R* is oil recycle ratio, %; *W*_*oe*_ is the quality of extracted oil (g); *P*_*oe*_ is the content of extracted oil, %; *W*_*om*_ is the weight of waste drilling fluid (g); *P*_*om*_ is the oil content of waste drilling fluid, %.

Five kinds of waste oil-based drilling fluid were disposed of in the same way above to calculate the rate of oil removal. The related data and the result are shown in Table 5.

As the date in Table 5 showed, the switchable deoiling agent has stably good effect to dispose of waste oil-based drilling fluid. Oil contents of extracted oil are from 96.8% to 97.8%, and the average percentage is 97.2%. Oil recycle ratios are from 93.7% to 94.2% and the average percentage is 93.9%.

The oil content of oily wastewater and oily solid waste was determined by infrared spectrophotometry. The samples were extracted and diluted according to the method described in Section 2.2.3. Oil content was detected with infrared oil content analyzer (Huaxia Kechuang Oil420). Consider

(4) $$\begin{array}{c} P_{w}=k\phi_{w},\\ P_{s}=\frac{k\phi_{s}}{\rho_{w}}, \end{array}$$

where *P*_*w*_ is oil content of oily wastewater (mg/L); *K* is dilution multiple; *φ*_*w*_ is the oil content of diluted waste water determined by infrared spectrophotometry (mg/L); *P*_*s*_ is oil content of oily solid waste, %; *φ*_*s*_ is the oil content of diluted water extracted from waste solid determined by infrared spectrophotometry (mg/L); and *ρ*_*w*_ is the density of tetrachloromethane (g/mL).

Five kinds of waste oil-based drilling fluid were disposed of in the same way above; the results are shown in Table 6. As shown in this table, the oil content of wastewater is between 12.83% and 16.49%, and the average percentage is 14.51%. The oil content of oily solid waste is from 1.929% to 2.915%, and the average percentage is 2.443%. It indicates that the solid waste is harmless in China. Therefore, the treatment of the solid waste would be much easier than the treatment of the original waste oil-based drilling fluid [25, 26]. These results clearly show that SDA can significantly reduce the oil content of the oil-based drilling fluid.

### 3.3. Structural Characterization and Mechanism of Action

#### 3.3.1. Structural Characterization

**Figure 6:**
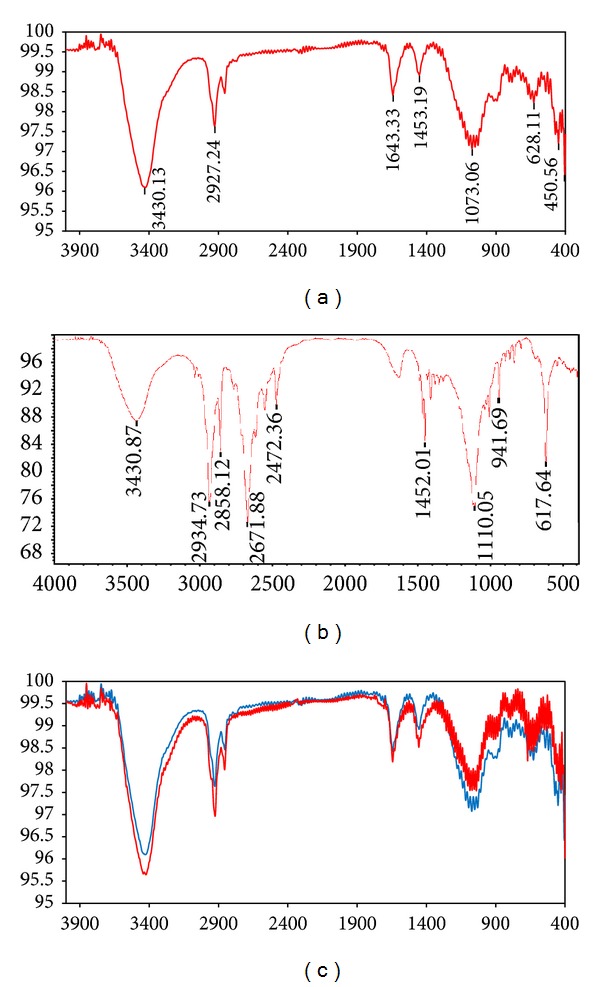
FTIR spectra of SDA.

Fourier transform infrared spectroscopy was used to analyze the structure of composite product (Figure 6). The location of the absorption peaks and corresponding transmittance were found after the data from spectrogram were processed.

Figure 6 shows the infrared spectroscopies of switchable deoiling agent in three stages of the switching progress.  Figure 6(a) shows the infrared spectroscopy of original SDA, which is hydrophobic; Figure 6(b) shows the infrared spectroscopy of hydrophilic SDA switched from original SDA; Figure 6(c) shows the comparison of the infrared spectroscopy of the original SDA and the infrared spectroscopy of hydrophobic SDA switched back from hydrophilic SDA.

In Figure 6(a), the adsorption peak at 1073.06 cm^−1^ has relationship with C–C bond. The two peaks at 1349 cm^−1^ and 1453.19 cm^−1^ come from C–H bonds in methyl. Another peak at 2927.24 cm^−1^ is observed as methyl, methylene, and methane. The emergence of peak at 3430.13 cm^−1^ means the existence of secondary amines or tertiary amines. According to the analysis results, the synthetic product contains large amounts of methyl, amine, and secondary carbon atoms. So it is speculated that synthetic product is based on one or several amines as the main component.

Comparison of Figures 6(a) and 6(b) shows that the position of absorption peak in Figure 6(a) is similar to the position of absorption peak is Figure 6(b). The main differences between these two figures are as follows: (1) in Figure 6(b), there is an obvious absorption peak at 2671.88 cm^−1^, which means the emergence of ammonium cations; (2) in Figure 6(b), the absorption peak is pronounced weaken at 3430.87 cm^−1^, which means the reduction of tertiary amine functional group. Therefore, the main change in the progress of switching hydrophobic SDA to hydrophilic SDA is that the tertiary amine is converted to ammonium salt with the effect of CO_2_ (gas A) and water.

In Figure 6(c), the red curve is the infrared spectroscopy of the original SDA which is similar to the infrared spectroscopy in Figure 6(a). The blue curve is the infrared spectroscopy of the recycled SDA switched from hydrophilic SDA by passing gas B through. The blue curve shows high similarity to the red curve, which means that the recycled SDA has little difference with the original SDA and can be reused.

#### 3.3.2. Mechanisms of Action

It is the main function for switchable deoiling agent, which has the characteristic of controllable hydrophilic transformation, to remove and recycle oil in the waste oil-based drilling fluid. Its main action principle is composed of deoiling mechanism and transformation mechanism.

The deoiling mechanism is based on the principle of the dissolution in the similar material structure. It refers to the similar structure and intersolubility of the solute and solvent, which means that in this paper the polar solutes dissolve in polar solvents while nonpolar solutes dissolve in nonpolar solvents. Waste oil-based drilling fluid is emulsion composed of water, oil, and solid impurities and so on. For the reason that the oil phase is mainly nonpolar and the water is polar, the oil phase is easily extracted by nonpolar solvents. And, in general, deoiling agent, which is hydrophobic, can absorb oil to realize oil-water separation. However, when gas A is passed into the mixture, oil is separated alone because deoiling agent has integrated with water for its change of hydrophilic nature. Similarly, after gas B is passed into the aqueous phase, deoiling agent is separated from water because it is hydrophobic again. So, the deoiling agent can be reused:

(5) NR3+H2O⇌+gas A+gas B  (−gas A)⁡R3NH++(OH−+gas  A)

The switching mechanism is that hydrophobic amine, the main composition of deoiling agent, can react reversibly according to the change of gas. As shown in the conversion mechanism (5), the hydrophobic amines (R is saturated alkyl) react with gas A to form hydrophilic product when gas A is passed into the aqueous phase. However, the reaction reverses to form original hydrophobic amine when gas B is passed into to replace gas A. Because of different reaction rate and activation energy the forward and converse reaction needs, there is great different conversion rate between the two reactions.

## 4. Conclusions

In summary, hydrophilicity of deoiling agent used for waste oil-based drilling fluid is convertible according to the need of human. Normally, deoiling agent is hydrophobic. However, it is hydrophilic when gas A (CO_2_) is passed into and it is hydrophobic again when gas B (air, Ar, or N_2_) is passed into. It is effective to use deoiling agent to deal with waste oil-based drilling fluid. The oil removal rate can reach 94% and the oil content of extracted oil is about 97%. The residues of deoiled waste oil-based drilling fluid are water phase and solid phase. The test result shows that the oil content of wastewater is below 17 mg/L and the oil content of oily solid waste is below 3%.

According to the analysis results of FTIR, the synthetic product contains large amounts of methyl, amine, and secondary carbon atoms. The amines especially are the leading parts. The deoiling mechanism of the switchable deoiling agent is based on the principle of the dissolution in the similar material structure. The oil is extracted from the waste oil-based drilling fluid based on the hydrophobicity of deoiling agent and the deoiling agent is recyclable for its switchable performance. The transition mechanism of it is mainly based on the reversible reaction among the amines with the water and gas. Thus, using SDA to treat the oil-based drilling waste fluid can recycle not only the oil in the fluid but also the SDA itself, which reduces waste of resource. Furthermore, with SDA, the waste oil-based drilling fluid can be treated without complex process, expensive equipment, and harsh conditions. Therefore, this technology can significantly reduce the waste of resources, energy, and the cost, comparing with the commonly used technologies.

The conversion process of deoiling agent is rapid from hydrophobic to hydrophilic and the oil loss during this process is negligible. However, the recovery process of deoiling agent, switch from hydrophilic to hydrophobic, is slow and the oil loss cannot be neglected in this process. Therefore, the next important research is how to improve the conversion rate from hydrophilic to hydrophobic and reduce the oil loss from hydrophilic to hydrophobic.

## Figures and Tables

**Table 1:** Content of each composition of waste oil-based drilling fluid.

Sample source	Water content (wt%)	Solidity content (wt%)	Oil content (wt%)
Unused oil-based drilling fluid (Daqing Oilfield)	58.8	14.5	26.7
Waste oil-based drilling fluid (number 501, W.S., Daqing Oilfield)	65.8	3.3	30.9
Displacing mud (number 501, W.S., Daqing Oilfield)	47.3	13.1	39.6
Waste oil-based drilling fluid (number 11, X., Daqing Oilfield)	50.4	20.4	29.2
Displacing mud (number 11, X., Daqing Oilfield)	44.3	19.2	36.5

**Table 2:** Main pollutants in waste oil-based drilling fluid.

Sample source	Cr (mg/kg)	Pb (mg/kg)	As (mg/kg)	Hg (mg/kg)	Cd (mg/kg)	Oil (mg/kg)
Unused oil-based drilling fluid (Daqing Oilfield)	18.50	14.74	14.30	1.239	0.14	267000
Waste oil-based drilling fluid (number 501, W.S., Daqing Oilfield)	10.70	21.20	20.38	0.754	0.35	309000
Displacing mud (number 501, W.S., Daqing Oilfield)	21.30	11.50	15.73	1.213	0.17	396000
Waste oil-based drilling fluid (number11, X., Daqing Oilfield)	13.60	30.70	17.92	0.836	0.41	292000
Displacing mud (number 11, X., Daqing Oilfield)	9.80	16.40	10.30	0.727	0.35	365000
*Control standards for pollutants in sledges from agricultural* (GB4284-84)	≤1000	≤1000	≤75	≤15	≤20	≤3000

**Table 3:** Relationship between volume change of all phases and time of passing gas through when the deoiling agent switches from hydrophobicity to hydrophilicity.

Time of passing gas through (min)	Volume of hydrophilic layer (mL)	Volume of hydrophobic layer (mL)	Switched volume (mL)	Conversion rate (%)
0	10	10	0	0
2.5	12.6	7.4	2.6	26
5	15	5	5	50
7.5	17.4	2.6	7.4	74
10	20	0	10	100

**Table 4:** Relationship between recovery rate of SDA and time of passing gas through when the deoiling agent switches from hydrophilicity to hydrophobicity.

Gas injection time (min)	Volume of hydrophobic layer (mL)	Recovery rate (%)
0	0	0
20	1	10
40	2.5	25
60	3.9	39
80	5.1	51
100	6.4	64
120	6.5	65
140	6.5	65

The highest recovery rate is 65%. Therefore, 35% SDA is lost in the process of switching. Such high loss rate may be due to long blowing.

**Table 5:** The treatment result of five kinds of waste oil-based drilling fluid.

Number	Weight of samples (g)	Oil content of samples (%)	Weight of extracted oil (g)	Oil content of extracted oil (%)	Oil recycle ratio (%)
1	41.5	26.7	10.68	97.3	93.8
2	35.1	30.9	10.53	96.9	94.1
3	27.4	39.6	10.40	97.8	93.7
4	36.7	29.2	10.40	97.1	94.2
5	30.1	36.5	10.64	96.8	93.7
Average	**—**	**—**	**—**	**97.2**	**93.9**

**Table 6:** The oil content of oily wastewater and oily solid waste.

Number	The measured value of water (mg/L)	The measured value of solid (mg/L)	Oil content of oily wastewater (mg/L)	Oil content of oily solid waste (%)
1	73.525	33.384	14.71	2.616
2	82.437	24.617	16.49	1.929
3	66.553	37.194	13.31	2.915
4	75.968	28.752	15.19	2.253
5	64.142	31.931	12.83	2.502
Average	**—**	**—**	**14.51**	**2.443**
